# *Adenophora Stricta* Root Extract Alleviates Airway Inflammation in Mice with Ovalbumin-Induced Allergic Asthma

**DOI:** 10.3390/antiox12040922

**Published:** 2023-04-13

**Authors:** Cheol-Jong Jung, Seok-Man Park, Dae-Geon Lee, Yeong-Eun Yu, Tae-Hun Ku, Im-Joung La, Il-Je Cho, Sae-Kwang Ku

**Affiliations:** 1 Department of Histology and Anatomy, College of Korean Medicine, Daegu Haany University, Gyeongsan 38610, Republic of Korea; oc_cjjung@okchundang.co.kr (C.-J.J.); smpark@okchundang.co.kr (S.-M.P.); ghost71715@okchundang.co.kr (D.-G.L.); 2 Central Research Center, Okchundang Inc., Daegu 41059, Republic of Korea; youye@okchundang.co.kr; 3 Okchundang Korean Medicine Clinic, Ulsan 44900, Republic of Korea; oc100002@okchundang.co.kr; 4 Atomy R&D Center, Gongju 32511, Republic of Korea; imjna@atomy.kr

**Keywords:** *Adenophora stricta* root extract, asthma, lipopolysaccharide, ovalbumin, Raw264.7 macrophage cells

## Abstract

*Adenophora stricta* Miq. (*Campanulaceae* family) is a traditional herb used for relieving cough and phlegm in East Asia. This study explored the effects of *A. stricta* root extract (AsE) in ovalbumin (OVA)-induced allergic asthma and lipopolysaccharide (LPS)-stimulated macrophages. Administration of 100–400 mg/kg AsE dose-dependently decreased pulmonary congestion and suppressed the reduction of alveolar surface area in mice with OVA-mediated allergic asthma. Histopathological analysis of lung tissue and cytological analysis of bronchioalveolar lavage fluid showed that AsE administration significantly attenuated inflammatory cell infiltration into the lungs. In addition, AsE also alleviated OVA-specific immunoglobulin E, interleukin (IL)-4, and IL-5 production, which are essential for OVA-dependent activation of T helper 2 lymphocytes. In Raw264.7 macrophage cells, AsE significantly blocked nitric oxide, tumor necrosis factor-α, IL-1β, IL-6, and monocyte chemoattractant factor-1 production in response to LPS. Results from an immunoblot assay revealed that AsE inhibited the phosphorylation of c-jun N-terminal kinase, inhibitory-κB kinase α/β, and p65 in LPS-stimulated cells. Furthermore, 2-furoic acid, 5-hydroxymethylfurfural, and vanillic acid 4-β-D-glucopyranoside in AsE were shown to inhibit the production of proinflammatory mediators by LPS. Taken together, the present results suggest that *A. stricta* root will be a useful herb for relieving allergic asthma through managing airway inflammation.

## 1. Introduction

Asthma is a chronic airway inflammatory disease with respiratory symptoms, such as coughing, wheezing, shortness of breath, and chest tightness [1]. The prevalence of asthma is continuously increasing and it is known that approximately 262 million people worldwide suffered from asthma in 2019 [2]. Although asthma is now recognized as one of the representative heterogeneous diseases, according to the differences between patients (e.g., age of onset, severity, comorbidity, and response to treatment) [3], it is estimated that more than half of asthma patients have type 2-high endotype [4]. In type 2-high asthma, various allergens stimulate the airway epithelial cells to produce alarmins (e.g., interleukin (IL)-33, IL-25, and thymic stromal lymphopoietin) for facilitating the interaction between conventional dendritic cells and T helper 2 (Th2) lymphocytes in the draining nodes. Then, Th2 cytokines (e.g., IL-4 and IL-5) from activated T cells promote the secretion of allergen-specific immunoglobulin (Ig) E in plasma cells, allow extravasation of eosinophils, and accelerate mucus production. The vicious cycle between repeated allergen exposure and Th2-dependent inflammation narrows the diameter of the airway lumen and triggers bronchial hyperresponsiveness [1,5]. Albeit inhaled corticosteroids in combination with long-acting β-agonists being prescribed as standard drugs for controlling asthma [6], they cannot be used for long periods of time due to side effects. In addition, the ultimate goal of asthma treatment is only to manage inflammation to avoid exacerbation of the disease progression, because asthma cannot be cured. Therefore, medical herbs that possess potent anti-inflammatory activity without serious side effects have been extensively studied as alternative and complementary options to prevent asthma and to reduce clinical symptoms associated with asthma [7].

Of diverse potential herbs, Radix Adenophorae, dried root of *Adenophora stricta* Miq. or *A. triphylla* var. *japonica* (Regel.) Hara of the *Campanulaceae* family, is a traditional antitussive and expectorant herbal drug that replenishes energy to the lungs and reduces fever in the lungs [8,9]. Throughout modern ethnopharmacological study, it has been proven that administration of *A. triphylla* blocks coughing caused by inhalation of ammonium hydroxide and enhances mucous secretion [10]. In addition, certain triterpenoids isolated from *A. triphylla* have been reported to inhibit the expression of mucin 5AC in airway epithelial cells [11]. Moreover, *A. triphylla* blocks *Candida* biofilm formation, attenuates weight gain and metabolic disorders in mice fed a high-fat diet, and suppresses the proliferation of lung and gastric cancer cells [12,13,14,15]. In contrast, regarding *A. triphylla*, little has been known about the pharmacological efficacy of *A. stricta*, except for estrogenic activity. Based on the results from our preliminary screening study that *A. stricta* exhibited a better pulmonary protective effect than *A. triphylla*, we recently reported that *A. stricta* root extract (AsE), which contains about 15 mg/g flavonoids as baicalein equivalent, is a useful medicinal herb to alleviate the lung damage and mucus stasis induced by the inhalation of fine particulate matter less than 2.5 μm [16]. Moreover, we also demonstrated that AsE is capable of protecting the lung epithelial cells from oxidative stress via inducing nuclear factor erythroid 2-related factor 2-dependent antioxidant genes [16]. In order to expand our understanding of the efficacy of *A. stricta* based on traditional knowledge, and to use *A. stricta* as a nutraceutical for promoting human health, we would like to further validate the beneficial effects of *A. stricta* in other types of lung injury models. In this regard, the present study investigated the pulmonary protective effect of AsE in ovalbumin (OVA)-induced allergic asthma. In addition, we further explored the anti-inflammatory potential of AsE in macrophages and sought to elucidate the molecular mechanisms involved.

## 2. Materials and Methods

### 2.1. Animal Experiment and Drug Treatment

*Adenophora stricta* root extract (AsE) was prepared by spray-drying an aqueous extract of the *A. stricta* root, as previously described [16]. After receiving research ethics approval for the protocol from the Institutional Animal Care and Use Committee of Daegu Haany University (approval no., DHU2022-029), the animal experiment was conducted to induce OVA-mediated allergic asthma, as previously reported [17]. C57BL/6JBomTac (C57BL/6) mice (gender, female; age, 6 weeks old) (Daehan Bio Link Co.; Eumsung, Republic of Korea) were randomly allocated into 6 groups (*N* = 10 per group): vehicle, OVA, OVA + DEXA, OVA + AsE-400, OVA + AsE-200, and OVA + AsE-100. For sensitizing immune response, OVA (5 mg/kg) (Sigma-Aldrich; St. Louise, Mo, USA) and aluminum hydroxide gel (0.45 mg/kg; Sigma-Aldrich) dissolved in saline were intraperitoneally injected on days 0 and 7 (i.e., day 0, the first day injecting OVA). For boosting allergic asthma, OVA (10 μg in 50 μL saline) alone was intranasally administered twice on days 14 and 15. AsE (100–400 mg/kg) dissolved in distilled water was orally administered once a day from day 0 to day 15. Instead of AsE, a dexamethasone–water soluble (DEXA; 0.75 mg/kg) (Sigma-Aldrich) was orally administered. On days 0, 1, 14, and 15, either AsE or DEXA was administered 1 h after challenging OVA. Mice in the vehicle group received an intraperitoneal injection of OVA without aluminum hydroxide, and were intranasally challenged with saline and orally administered distilled water, respectively. For subsequent analysis, the blood and lungs were collected from euthanized mice 1 day after the last drug administration.

### 2.2. Measurement of Body and Lung Weight

Body weight was measured on days −1, 0, 1, 7, 14, 15, and 16 using a balance (XB320M, Precisa Instrument; Zürich, Switzerland). Body weight gain was calculated by subtracting the day 0 weight from day 16, and the relative lung weight was calculated by dividing the lung weight by the body weight on day 16.

### 2.3. Histopathological Analyses

After images were captured in the left lung, the congested region was calculated as the percentage of congested area using an automated image analyzing software (*i*Solution FL 9.1, IMT *i*-solution Inc.; Bernaby, BC, Canada). Then, left lateral lobes were fixed, embedded in paraffin, cut and stained with hematoxylin and eosin, Congo red or toluidine blue, as previously described [17]. The secondary bronchus was observed under Eclipse 80*i* light microscope (Nikon; Tokyo, Japan), and the alveolar surface area (%/mm^2^ of lung tissue), thickness of alveolar septum (μm), infiltrated eosinophils (cell number/mm^2^ of lung tissue), and infiltrated mast cells (cell number/mm^2^ of lung tissue) in the lung sections were counted using an automated image analyzing software (*i*Solution FL 9.1).

### 2.4. Enzyme-Linked Immunosorbent Assay

Serum was isolated by conventional centrifugation of the blood (e.g., 12,000× *g*, 10 min, 4 °C). In addition, bronchoalveolar lavage fluid (BALF) was obtained by injecting 1 mL of saline after the left secondary bronchus and right lower secondary bronchus were ligated using two MB324 nylon threads (AILEE; Pusan, Republic of Korea), and the supernatant was collected by centrifugation of BALF (e.g., 3000× *g*, 5 min, 4 °C). Moreover, the conditioned medium collected from treated Raw264.7 cells was clarified by centrifugation (e.g., 3000× *g*, 5 min, 4 °C). Levels of OVA-specific immunoglobulin E (IgE) (Mybiosource; San Diego, CA, USA) in serum, interleukin (IL)-4 (Mybiosource) and IL-5 (Mybiosource) in BALF supernatant, and prostaglandin E_2_ (PGE_2_) (R&D Systems; Minneapolis, MN, USA), tumor necrosis factor-α (TNF-α) (BD Biosciences; San Diego, CA, USA), IL-1β (BD Biosciences), IL-6 (BD Biosciences), and monocyte chemoattractant protein-1 (MCP-1) (BD Biosciences) were measured in a clarified conditioned medium, according to the supplier’s instructions.

### 2.5. Cytological Analyses of Bronchoalveolar Lavage Fluid 

BALF was stained with trypan blue (Sigma-Aldrich) for counting the total cell number using a Countess C10281 Automated Cell Counter (Invitrogen; Carlsbad, CA, USA). In addition, total leukocytes, lymphocytes, neutrophils, eosinophils, and monocytes were counted using a Cell-DYN^®^ 3700 Hematology Analyzer (Abbott Laboratories; Abbott Park, IL, USA).

### 2.6. Real-Time Polymerase Chain Reaction

Lung lower right lobe or Raw264.7 cells were homogenized with the addition of Trizol (Invitrogen) or Tri Reagent (Bioscience Technology; Daegu, Republic of Korea) using a Taco^TM^ Prep Bead Beater (GeneReach Biotechnology; Taichung, Taiwan), and total RNA was extracted according to the manufacturer’s instructions. cDNA was obtained by reacting RNA (2 μg) with the AccuPower^®^ RT Premix (Bioneer; Daejeon, Republic of Korea). Real-time polymerase chain reaction (PCR) was conducted using TB Green^®^ Premix Ex Taq (Takara; Shiga, Japan) and the CFX96 Real-Time PCR Detection System (Bio-Rad; Hercules, CA, USA). Glyceraldehyde 3-phosphate dehydrogenase was used as a housekeeping gene, and the relative mRNA level of specific genes was quantified based on the ΔΔC_T_ method [18]. The following primers synthesized by Bioneer were used to amplify specific murine genes: IL-4 (GenBank accession #, NM_021283.2; amplicon size, 385 bp), 5′-GAATGTACCAGGAGCCATATC-3′ (forward), 5′-CTCAGTACTACGAGTAATCCA-3′ (backward); IL-5 (GenBank accession #, NM_010558.1; amplicon size, 201 bp), 5′-TCACCGAGCTCTGTTGACAA-3′ (forward), 5′-CCACACTTCTCTTTTTGGCG-3′ (backward); inducible nitric oxide synthase (iNOS) (GenBank accession #, NM_010927.4; amplicon size, 325 bp), 5′-GACAAGCTGCATGTGACATC-3′ (forward), 5′-GCTGGTAGGTTCCTGTTGTT-3′ (backward); glyceraldehyde 3-phosphate dehydrogenase (GenBank accession #, NM_001289726.1; amplicon size, 141 bp), 5′-AACGACCCCTTCATTGAC-3′ (forward), 5′-TCCACGACATACTCAGCAC-3′ (backward).

### 2.7. Cell Culture and Drug Treatment

Raw264.7 cells (murine macrophage-derived cell line) were obtained from the American Type Culture Collection (Rockville, MD, USA) and maintained under Dulbecco’s modified eagle’s medium (WEL GENE; Gyeongsan, Republic of Korea), with 10% fetal bovine serum (WEL GENE) and 1% antibiotic antimycotic solution (WEL GENE). Raw264.7 cells grown in an appropriate multiwell plate were changed to a serum-free medium, pretreated with 1 or 3 mg/mL AsE for 1 h, and then exposed to 1 μg/mL lipopolysaccharide (LPS) for 1–18 h.

### 2.8. Measurement of Nitric Oxide Production and Cell Viability

After treatment with LPS for 18 h, the conditioned medium was collected, clarified, and reacted with Griess reagent. To quantify the nitric oxide contained in the conditioned medium, absorbance at 540 nm wavelength was measured using a Synergy HTX plate reader (BioTek; Winooski, VT, USA). Nitric oxide production was expressed as a relative ratio to the absorbance of the control cell. In addition, cells were further reacted with 0.5 mg/mL thiazolyl blue tetrazolium bromide (Sigma-Aldrich) for 4 h, and formazan crystals generated from the viable cells were solubilized by adding dimethyl sulfoxide. After measuring optical intensity at 570 nm, the relative cell viability was expressed as a percentage of the absorbance of the control cell.

### 2.9. Protein Extraction and Immunoblot Analyses

Primary antibodies for phosphorylated c-jun N-terminal kinase (JNK; Thr^183^/Tyr^185^), JNK, phosphorylated p38 (Thr^180^/Tyr^182^), p38, phosphorylated extracellular signal-regulated kinase (ERK; Thr^202^/Tyr^204^), ERK, phosphorylated inhibitory-κBα (I-κBα; Ser^32^), phosphorylated I-κB kinase α/β (IKKα/β; Ser^176/180^), IKKα, phosphorylated p65 (Ser^536^), and histone H3, as well as horseradish peroxidase-conjugated secondary antibodies were provided by Cell Signaling Technology (Danvers, MA, USA). Antibodies for I-κBα and p65 were obtained from Santa Cruz Biotechnology (Santa Cruz, CA, USA), and an anti-β-actin antibody was from Sigma-Aldrich. Preparation of whole-cell lysates and nuclear extracts, protein quantification, sodium dodecyl sulfate polyacrylamide gel electrophoresis, transfer of the separated proteins to the nitrocellulose membrane, and antibody incubation were performed, as previously described [19]. Chemiluminescence emitted from the membrane was detected using either Imager 600 (GE Healthcare Life Sciences; Little Chalfont, UK) or Fusion FX7 (Vilber Mourmat; Marne-la-Vallée, France). Densitometric analyses were conducted using the ImageJ software (https://imagej.nih.gov/ij; accessed on 12 June 2020).

### 2.10. Simultaneous Analysis of 2-Furoic Acid, 5-Hydroxymethylfurfural, and Vanillic Acid 4-β-D-glucopyranoside in AsE

Standard chemicals for 2-furoic acid (FA), 5-hydroxymethylfurfural (HMF), and vanillic acid 4-β-D-glucopyranoside (VA-4G) were supplied by Sigma-Adrich. AsE dissolved in water (0.04 g/mL) was additionally extracted for 30 min by ultrasonication (JAC-5020, Kodo Technical Research Co.; Hwasung, Republic of Korea). After filtering AsE using a 0.45 μm polyvinylidene difluoride syringe filter (Thermo Fisher Scientific; Waltham, MA, USA), the resulting solution was loaded onto Alliance e2695 high-performance liquid chromatography (HPLC) system (Waters; Milford, MA, USA) equipped with CAPCELL PAK ADME–HR C18 column (size 4.6 × 250 mm; pore size, 5 μm)(OSAKA SODA Co.; Osaka, Japan) and Waters 2998 photodiode array detector. AsE was eluted under a binary solution, comprising 0.05% trifluoroacetic acid (solution A) and 100% acetonitrile (solution B), and the mobile phase was changed from 100%:0% to 75%:25% (solution A: solution B) over a separation period of 60 min. The column temperature was 30 °C, and eluants were detected at a 254 nm wavelength. The concentrations of FA, HMF, and VA-4G in AsE were quantified by interpolating the peak areas showing the same retention time as the three compounds to each standard curve.

### 2.11. Statistical Analyses

Numerical results are expressed as the mean ± standard deviation, and a one-way analysis of variance test or Welch’s test was conducted to compare the means. The means between different experimental groups were further analyzed using either Tukey’s honestly significant difference test or Dunnett’s T3 test as post hoc analyses. Analyses were performed using SPSS Statistics 18 (SPSS Inc.; Chicago, IL, USA), and *p*-values below 0.05 were decided to be significant differences.

## 3. Results

### 3.1. Adenophora Stricta Root Extract Reduces Pulmonary Congestion in Mice with Ovalbumin-Mediated Allergic Asthma

**Figure 1:**
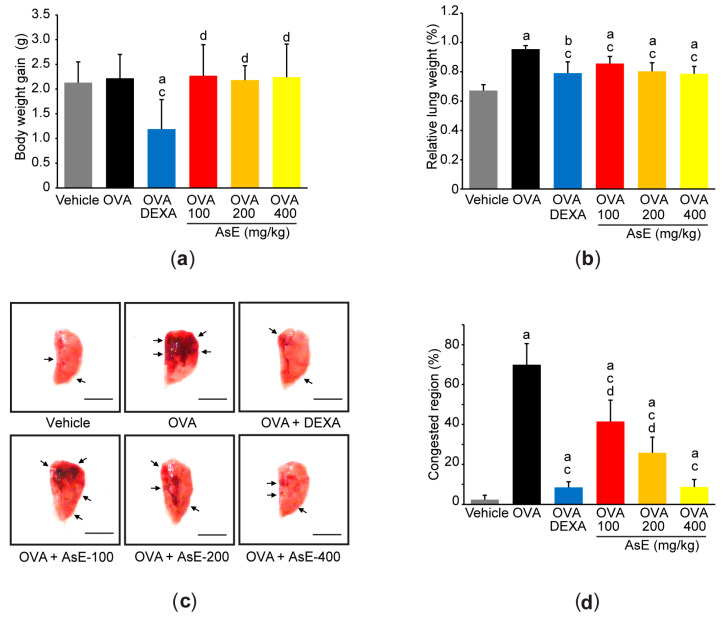
AsE reduces pulmonary congestion in OVA-induced allergic asthma. Body weight gain (**a**) and relative lung weight (**b**) were calculated as described in the Materials and Methods section. (**c**) Representative image of the left lung lobe. Arrows indicate the congested regions, and scale bars indicate 6.0 mm. (**d**) The congested region was calculated as the percentage of the congested area in the left lung. ^a^ *p* < 0.01, ^b^ *p* < 0.05 versus vehicle group; ^c^ *p* < 0.01 versus OVA group; ^d^ *p* < 0.01 versus OVA + DEXA group; AsE, *Adenophora stricta* root extract; DEXA, dexamethasone; OVA, ovalbumin.

Allergic asthma was induced in C57BL/6 mice by intraperitoneal injection of OVA with aluminum hydroxide on days 0 and 7, and an intranasal instillation of OVA on days 14 and 15. AsE (100–400 mg/kg) was orally administered once a day from days 0 to 15, and DEXA (0.75 mg/kg) was used as a reference drug. On day −1, there was no difference in the body weight among experimental groups. When body weights were measured on days 0, 1, 7, 14, 15, and 16, there were also no differences in the body weight between vehicle, OVA, and OVA + AsE groups. However, DEXA significantly reduced the body weight from day 7 onward, which was parallel with previous reports that DEXA reduces body weight probably due to muscle atrophy [16,17,20,21]. Consistently, there were no differences in the body weight gains between the vehicle, OVA, and OVA + AsE groups during the entire experimental period, and DEXA significantly decreased the body weight gain (Figure 1a). When the lungs of euthanized mice were weighed, OVA significantly increased the relative lung weight. However, oral administration of 100–400 mg/kg AsE and DEXA for sixteen consecutive days significantly prevented the OVA-mediated increase in relative lung weight. There were no differences in relative lung weight between OVA + AsE and OVA + DEXA groups (Figure 1b). Moreover, gross inspection of lung tissue showed that AsE dose-dependently reduced pulmonary congestion induced by OVA. The extent of reduction in the congested lung area in mice dosed with 400 mg/kg AsE was similar to that of mice dosed with DEXA (Figure 1c,d).

### 3.2. Adenophora Stricta Root Extract Mitigates Ovalbumin-Mediated Pulmonary Injury by Inhibiting Infiltration of Inflammatory Cells 

**Figure 2:**
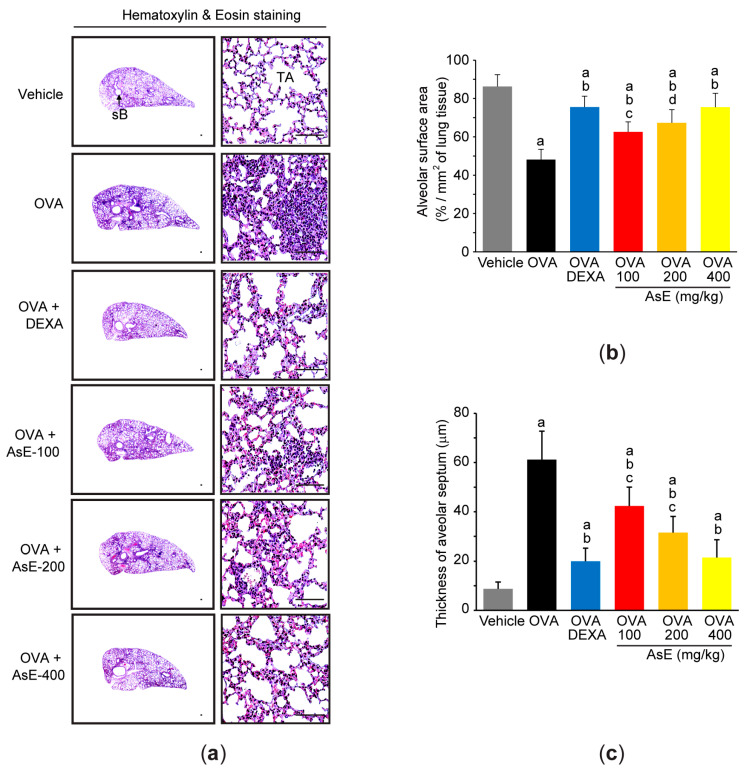
AsE mitigates pulmonary injury in OVA-induced allergic asthma. (**a**) Representative histological image of hematoxylin- and eosin-stained lung tissue. Scale bars indicate 200 μm. Alveolar surface area (**b**) and thickness of alveolar septum (**c**) were measured using an image analyzer. ^a^ *p* < 0.01 versus vehicle group; ^b^ *p* < 0.01 versus OVA group; ^c^ *p* < 0.01, ^d^ *p* < 0.05 versus OVA + DEXA group; sB, secondary bronchus; TA, terminal respiratory bronchiole alveoli.

To evaluate the effects of AsE on OVA-mediated pulmonary injury, lung tissues were stained with hematoxylin and eosin. Histopathological observation of lung tissue revealed that OVA challenge obviously shrank the alveolar surface area (Figure 2a,b) and enlarged the alveolar septum (Figure 2a,c). However, administration of three different doses of AsE significantly reduced the aforementioned pathological changes associated with asthma, and the lung protective effect by 400 mg/kg AsE was similar to that by DEXA (Figure 2b,c).

**Figure 3:**
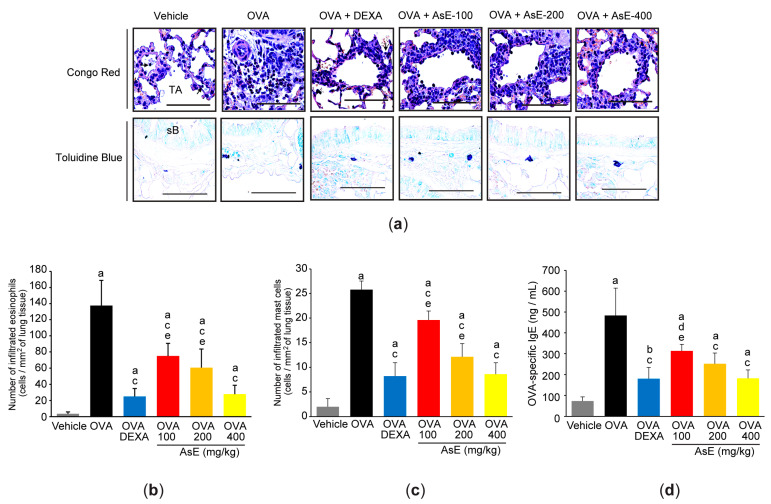
AsE inhibits the infiltration of inflammatory cells in the lungs. (**a**) Representative histological images of Congo-red- or toluidine-blue-stained lung tissue. Scale bars indicate 200 μm. Numbers of infiltrated eosinophils (**b**) and mast cells (**c**) were counted in the tissue sections stained with Congo red and toluidine blue, respectively. (**d**) Level of OVA-specific IgE was measured in the serum. ^a^ *p* < 0.01, ^b^ *p* < 0.05 versus vehicle group; ^c^ *p* < 0.01, ^d^ *p* < 0.05 versus OVA group; ^e^ *p* < 0.01 versus OVA + DEXA group; IgE, immunoglobulin E.

Next, lung tissues were specifically stained either with Congo red or toluidine blue to explore the effect of AsE on infiltrated inflammatory cells. OVA significantly increased the number of positive cells stained with Congo red (e.g., eosinophils) (Figure 3a,b) or toluidine blue (e.g., mast cells) (Figure 3a,c) in the lung tissue. Simultaneously, AsE dose-dependently inhibited the infiltration of Congo-red- and toluidine-blue-positive cells in the lungs (Figure 3b,c). Moreover, cytological analysis of BALFs confirmed that AsE could inhibit OVA-induced infiltration of immune cells, including total leukocytes, lymphocytes, neutrophils, eosinophils, and monocytes (Table 1). Furthermore, the production of OVA-specific IgE in the blood was also reduced in mouse groups given the three different doses of AsE (Figure 3d). In mice administered 400 mg/kg AsE, the inhibitory effects of the bronchoalveolar infiltration of immune cells and OVA-specific IgE production were comparable to those in mice administered DEXA (Figure 3 and Table 1).

### 3.3. AsE Attenuates OVA-Induced IL-4 and IL-5 Production in the Lungs 

**Figure 4:**
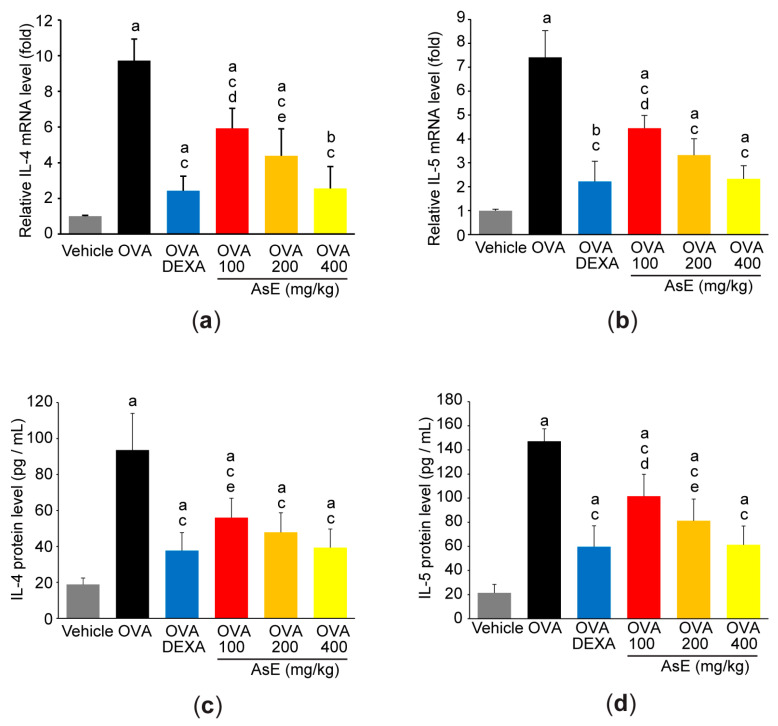
AsE attenuates IL-4 and IL-5 production in OVA-induced allergic asthma. mRNA levels of IL-4 (**a**) and IL-5 (**b**) in the lower right lobe of the lung were quantified by real-time PCR. In addition, IL-4 (**c**) and IL-5 (**d**) proteins in BALF were quantified by ELISA. ^a^ *p* < 0.01, ^b^ *p* < 0.05 versus vehicle group; ^c^ *p* < 0.01 versus OVA group; ^d^ *p* < 0.01, ^e^ *p* < 0.01 versus OVA + DEXA group; BALF, bronchoalveolar lavage fluid; ELISA, enzyme-linked immunosorbent assay; IL, interleukin; PCR, polymerase chain reaction.

Because IL-4 and IL-5 are representative Th2 cytokines that initiate chronic airway inflammation [1,5], we further investigated the levels of these cytokines in the lungs. As expected, OVA significantly increased mRNA expression of IL-4 and IL-5 in lung tissue (Figure 4a,b), as well as the protein levels of these cytokines in BALFs (Figure 4c,d). However, administration of three different doses of AsE suppressed the increase in the production of these cytokines in response to OVA. In addition, there were no statistical differences in IL-4 and IL-5 mRNA and protein levels between the groups of mice given 400 mg/kg AsE and DEXA (Figure 4).

### 3.4. Adenophora Stricta Root Extract Suppresses Proinflammatory Response in Raw264.7 Cells

**Figure 5:**
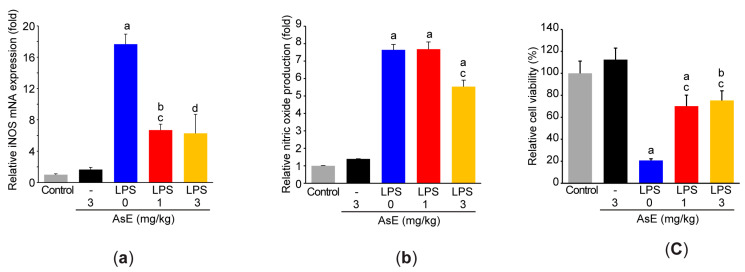
AsE suppresses nitric oxide production in LPS-stimulated Raw264.7 cells. (**a**) Real-time PCR. The mRNA level of iNOS was quantified after the AsE (1 or 3 mg/mL; for 1 h)-pretreated Raw264.7 cells were exposed to the LPS (1 μg/mL) for 6 h. Nitric oxide production (**b**) and cell viability (**c**) were measured after treating the cells with AsE (1 or 3 mg/mL; for 1 h) and LPS (1 μg/mL; for 18 h). ^a^ *p* < 0.01, ^b^ *p* < 0.05 versus control cells; ^c^ *p* < 0.01, ^d^ *p* < 0.01 versus LPS-treated cells; LPS, lipopolysaccharide; iNOS, inducible nitric oxide synthase.

Raw264.7 cells were adopted to explore molecular mechanisms associated with AsE-dependent inhibition of airway inflammation. As expected, LPS stimulation (1 μg/mL) in Raw264.7 cells significantly accumulated nitric oxide to the medium by upregulating iNOS mRNA (Figure 5a,b). However, AsE pretreatment significantly inhibited the increase in iNOS gene transcription and nitric oxide production by the LPS. AsE alone (3 mg/mL) had no effect on iNOS mRNA levels, as well as nitric oxide production (Figure 5a,b). To rule out the possibility that AsE could inhibit nitric oxide production through cytotoxicity against Raw264.7 cells, we further tested the effect of AsE on cell viability. As previously reported [19], the LPS reduced the viability of Raw264.7 cells. AsE in the presence of LPS significantly prevented the decrease in cell viability to LPS, and AsE alone had no effect on the cell viability (Figure 5c), suggesting that AsE inhibits NO production, irrespective of its cytotoxicity against Raw264.7 cells.

**Figure 6:**
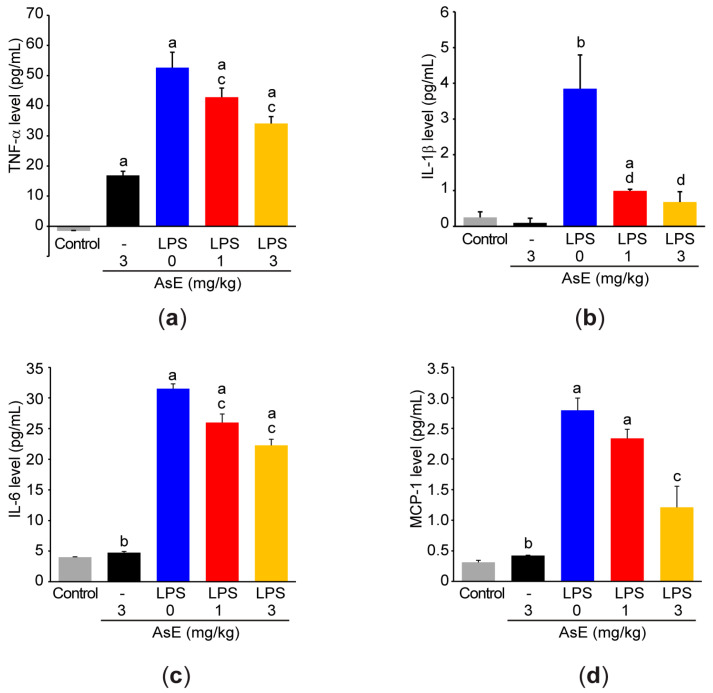
AsE reduces proinflammatory cytokine production in LPS-stimulated Raw264.7 cells. Raw264.7 cells were treated with AsE and LPS, as described in Figure 5b. Levels of TNF-α (**a**), IL-1β (**b**), IL-6 (**c**), and MCP-1 (**d**) were quantified in the conditioned medium. ^a^ *p* < 0.01, ^b^ *p* < 0.05 versus control cells; ^c^ *p* < 0.01, ^d^ *p* < 0.01 versus LPS-treated cells; MCP-1, monocyte chemoattractant protein-1; TNF-α, tumor necrosis factor-α.

Consistent with the results of iNOS mRNA, our additional ELISA results revealed that AsE also inhibited LPS-mediated induction of TNF-α, IL-1β, IL-6, and MCP-1 in a dose-dependent manner (Figure 6a–d).

### 3.5. Adenophora Stricta Root Extract Blocks c-Jun N-Terminal Kinase and Nuclear Factor-κB Activations in Raw264.7 Cells

**Figure 7:**
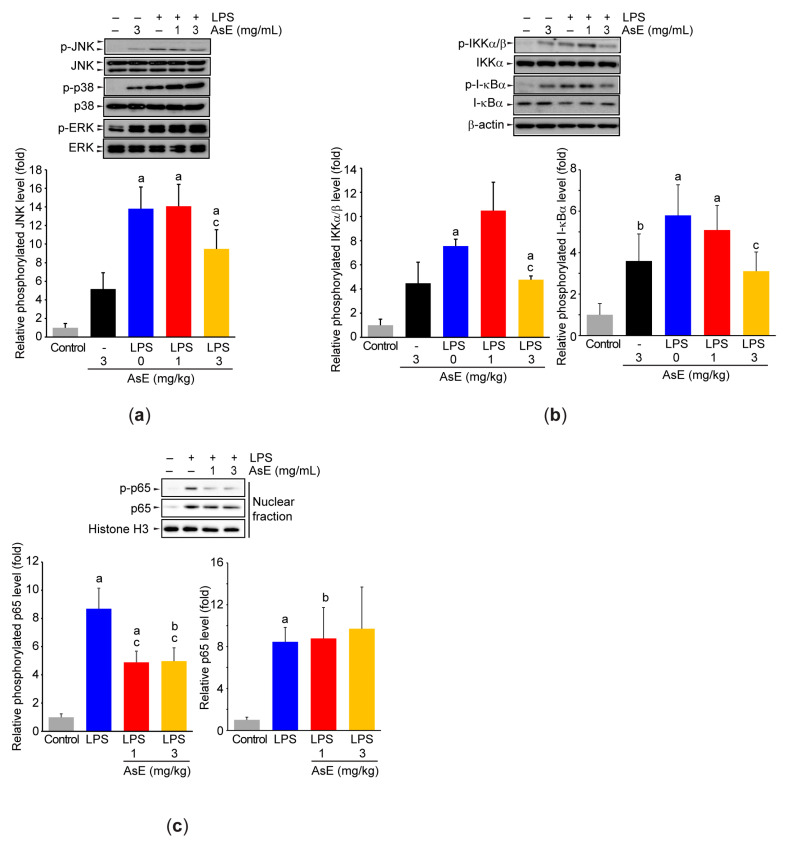
AsE blocks JNK and NF-κB signaling pathways in LPS-stimulated Raw264.7 cells. Whole-cell lysates (**a**,**b**) and nuclear fraction were prepared after AsE (1 or 3 mg/mL)-pretreated Raw264.7 cells were subsequently exposed to the LPS (1 μg/mL) for 1 h. Changes in mitogen-activated protein kinases (**a**) and NF-κB signaling molecules (**b**,**c**) were monitored by immunoblot analysis. Equal protein loading was verified by immunoblotting of unphosphorylated protein (for (**a**)), β-actin (for (**b**)), or histone H3 (for (**c**)). ^a^ *p* < 0.01, ^b^ *p* < 0.05 versus control cells; ^c^ *p* < 0.05 versus LPS-treated cells; ERK, extracellular signal-regulated kinase; p-ERK, phosphorylated ERK; I-κBα, inhibitory-κBα; p-I-κBα, phosphorylated I-κBα; IKK, I-κB kinase; p-IKK, phosphorylated IKK; JNK, c-jun N-terminal kinase; p-JNK, phosphorylated JNK; p-p65, phosphorylated p65.

Since mitogen-activated protein kinase (MAPKs) and nuclear factor-κB (NF-κB) are pivotal signaling pathways to accelerate inflammatory response [22], we further explored the effect of AsE on the activation of these signaling pathways in LPS-stimulated Raw264.7 cells. Our immunoblot analyses showed that the LPS increased the levels of phosphorylated JNK, p38, and ERK. AsE pretreatment (3 mg/mL) significantly decreased JNK phosphorylation induced by the LPS, while AsE did not change the phosphorylation levels of p38 and ERK (Figure 7a). In addition, AsE (3 mg/mL) also inhibited IKKα/β and I-κBα phosphorylation in response to the LPS (Figure 7b). Although AsE did not alter LPS-dependent nuclear expression level of p65, a major subunit of NF-κB, AsE significantly suppressed the phosphorylation level of nuclear p65 (Figure 7c).

### 3.6. Two-Furoic Acid, 5-Hydroxymethylfurfural, and Vanillic Acid 4-β-D-Glucopyranoside in Adenophora Stricta Root Extract Are Bioactive Compounds for Regulating Inflammation in Raw264.7 Cells

**Figure 8:**
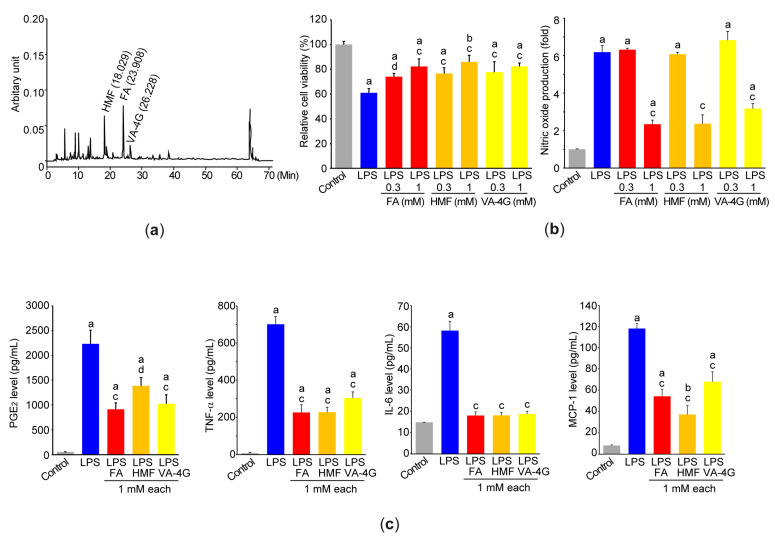
FA, HMF, and VA-4G are bioactive compounds responsible for the anti-inflammatory effect of AsE. (**a**) Representative HPLC chromatogram of AsE. Eluants were monitored at 254 nm, and the numbers in the chromatogram are retention times of each bioactive compound. (**b**) Effect of three bioactive compounds on nitric oxide production. Raw264.7 cells were pretreated with 0.3 and 1 mM of three bioactive compounds for 1 h, and subsequently exposed to the LPS for 18 h. Viable cells were determined by an MTT assay (**left**), and nitric oxide was measured by incubating conditioned medium with the Griess reagent (**right**). (**c**) Effect of three bioactive compounds on proinflammatory mediator production. Levels of proinflammatory mediators in conditioned medium were determined using commercial ELISA kits. ^a^ *p* < 0.01, ^b^ *p* < 0.05 versus control cells; ^c^ *p* < 0.01, ^d^ *p* < 0.01 versus LPS-treated cells; FA, 2-furoic acid; HMF, 5-hydroxymethyl furfural; HPLC, high-performance liquid chromatography; PGE_2_, prostaglandin E_2_; VA-4G, vanillic acid 4-β-D-glucopyranoside.

We recently reported that AsE contains vanillic acid 4-β-D-glucopyranoside (VA-4G) [16]. After column chromatography was additionally performed to purify other bioactive compounds of AsE, 2-furoic acid (FA) and 5-hydroxymethylfurfural (HMF) were further identified by nuclear magnetic resonance spectroscopy. Results from HPLC analysis indicated that the AsE used in the present study contained 208.39 ± 0.86 μg/g FA, 471.30 ± 4.57 μg/g of HMF, and 143.45 ± 1.08 μg/g of VA-4G (Figure 8a).

To elucidate the role of FA, HMF, and VA-4G on LPS-mediated inflammatory response, Raw264.7 cells were pretreated with 0.3 or 1 mM of these compounds, and then exposed to the LPS. The MTT assay showed that pretreatment with FA, HMF, or VA-4G significantly prevented the decrease in viability of Raw264.7 cells in response to the LPS (Figure 8b, left). Pretreatment with 0.3 mM of the three compounds had no effect on LPS-mediated nitric oxide production in a conditioned medium. However, pretreatment with three compounds at 1 mM concentration each significantly reduced the nitric oxide production by the LPS (Figure 8b, right). Moreover, pretreatment with FA, HMF, or VA-4G (1 mM each) also significantly suppressed the levels of PGE_2_, TNF-α, IL-6, and MCP-1 secreted into the culture medium by LPS stimulation (Figure 8c).

## 4. Discussion

The root of *A. stricta* has been reported to contain a number of bioactive compounds, such as benzoic acid derivatives (e.g., VA-4G, vanillic acid, vanillin, and syringic acid 4-O-β-D-glucopyranoside), phenols (e.g., p-hydroxybenzaldehyde, p-hydroxybenzoic acid, and p-hydroxyacetophenone), triterpenoids (e.g., methyl adenophorate, lupenone, 24-methylene cycloartenol, and sessilifolic acid), steroids (β-sitosterol and its derivatives), decursidin, and so on [23,24]. Among them, it has been reported that lupenone inhibits the expression of airway mucin in NCI-H292 cells [11]. In addition, β-sitosterol, vanillin, and vanillic acid can suppress airway inflammation [25,26,27]. In our previous study, we reported that VA-4G is a useful biomarker to manage the quality control of AsE [16]. Because glycoside linkages in most natural products can be broken by the acidic environment or commensal microorganisms in the gut, vanillic acid (aglycosylated metabolite of VA-4G) is probably produced after oral ingestion. Although there are no studies on the effects of VA-4G on human health, it has been reported that vanillic acid possesses diverse pharmacological activities for promoting human health [28,29]. Especially, vanillic acid has also been reported to mitigate lung injuries from toxic stimuli [26,30]. As part of continuous research on bioactive compounds responsible for lung protection, FA and HMF were additionally identified in AsE. In addition, we showed that FA, HMF, and VA-4G protected the Raw264.7 cells from LPS-induced cytotoxicity. Moreover, all three compounds are capable of reducing nitric oxide, PGE_2_, TNF-α, IL-6, and MCP-1 production in Raw264.7 cells stimulated with LPS. Albeit our results clearly showed that FA, HMF, and VA-4G were able to inhibit the proinflammatory response in Raw264.7 cells stimulated with the LPS, the concentrations of the three compounds contained in AsE appear to be too low to contribute to the AsE anti-inflammatory effect. Therefore, the aforementioned compounds, as well as other unidentified compounds in AsE, probably contribute to protecting the lungs from allergic inflammation.

Accumulated evidence from mice genetically lacking IL-4 and IL-5 suggest that these molecules are essential Th2 cytokines for provoking airway inflammation and alveolar hyperresponsiveness [1,31,32]. In the present study, we showed that AsE efficaciously inhibited IL-4 and IL-5 production in airway and lung tissues. During asthma progression, IL-4 induces an isotype switch to IgE in memory IgG-positive B lymphocytes. Then, IgE increases vascular permeability by stimulating the degranulation of mast cells and basophils, facilitates extravasation of inflammatory cells into lung tissue by increasing the expression of adhesion molecules, and leads to alveolar hyperresponsiveness of the airway smooth muscles [33,34]. In addition, IL-5 stimulates the maturation of inflammatory eosinophils (e.g., hypodense eosinophils in human) and their recruitment to the inflamed lungs [35], where the eosinophils disrupt lung architecture, exacerbate inflammation, and modulate the tone of parasympathetic and sensory nerves by secreting bioactive substances stored in their granules [36,37]. Therefore, AsE-mediated downregulation of these Th2 cytokines is likely one of the molecular mechanisms by which the AsE protects lung tissue through inhibiting immune cell infiltration in the mouse lungs of an OVA-induced asthma model.

Interaction between allergen-inhaled dendritic cells and Th2 lymphocytes, as well as innate immunity induced by type 2 innate lymphoid cells, basophils, mast cells, and macrophages also contribute to initiating and exacerbating asthma [1]. Clinical observations have especially shown that the proinflammatory cytokines mainly produced by macrophages (e.g., TNF-α, IL-1β, IL-6, and MCP-1) are increased in the BALF of patients with asthma [38,39,40]. In addition, studies have reported that chemical and genetic depletion of circulating monocytes abrogates lung inflammation [41,42], providing evidence that recruited macrophages are not just bystander, but active players contributing to the progression of allergic asthma. Although alveolar macrophages, which account for more than half of lung-resident immune cells, have long been thought to maintain homeostasis, primarily by suppressing inflammation [43], recent studies conversely suggest that a specific subset of alveolar macrophages also enhances the inflammatory response in lung tissue [44,45,46]. Regarding the importance of macrophage to airway inflammation, we further investigated the in vitro effect of AsE on LPS-stimulated Raw264.7 cells, and found that AsE had an ability to inhibit nitric oxide production as a result of reduced iNOS gene transcription. In addition, AsE dose-dependently suppressed the induction of proinflammatory cytokines in response to the LPS. Therefore, the present results suggest that the inhibition of macrophage-dependent innate immunity, as well as Th2-dependent adaptive immunity is probably correlated with the alleviation of airway inflammation and lung injury by AsE administration.

MAPKs and NF-κB are representative signaling molecules that are activated simultaneously in response to the LPS in macrophages [22]. After binding to the LPS, the cytoplasmic domain of Toll-like receptor 4 recruits the TIR-domain-containing adaptors (e.g., MyD88, TRIF, and TIRAP), and forms a protein complex. Then, the activated receptor facilitates the polyubiquitination of TRAF6 and results in the phosphorylation of TAK1, a member of MAPKKK. Finally, TAK1 facilitates the activation of several transcription factors via the phosphorylation of MAPK and IKK complex [22,47]. In the present study, we showed that AsE specifically inhibits the expression level of phosphorylated JNK, IKKα/β, I-κBα, and p65. JNK can phosphorylate a variety of substrates, including Jun, fos, and ATF, which comprise activator protein-1 transcription factor for the transactivation of diverse inflammatory genes [48]. In addition, JNK can activate NF-κB via an indirect regulation of IKK [49]. Moreover, TAK1-dependent IKK phosphorylation facilitates the activity of NF-κB at various stages. For instance, activated IKK can liberate NF-κB sequestered from the I-κBα complex through the degradation of phosphorylated I-κBα. Furthermore, IKK has also been reported as an upstream kinase that directly phosphorylates the transactivation domain of NF-κB (e.g., Ser^536^ of p65), and thereby enhances the transactivation potential NF-κB by promoting coactivator recruitment [50]. Because accumulated evidence indicates that the targeting of NF-κB and MAPK signaling molecules ameliorates allergic asthma in experimental animals [51,52], the inhibition of JNK and IKK phosphorylation by AsE probably contributes to attenuating airway inflammation.

## 5. Conclusions

The present results clearly showed that *Adenophora stricta* root extract (AsE) can protect the lung from allergic asthma by reducing the infiltration of inflammatory cells, as well as the production of T helper 2 lymphocyte-dependent cytokines. The reduction in airway inflammation by administration of AsE (400 mg/kg) was similar to that by administration of dexamethasone. In addition, AsE has an ability to decrease proinflammatory responses in macrophages by suppressing the phosphorylation of c-jun N-terminal kinase and inhibitory-κB kinase. Nevertheless, elucidation of pulmonary protective compounds in AsE, molecular targets of AsE during Th2-dependent immunity activation, the protective effect of AsE in asthma caused by other types of etiologies, toxicology of AsE, and AsE efficacy in human application are additionally needed to be further studied to reach a more general conclusion. If several remaining issues are appropriately addressed in the future, AsE will be a promising nutraceutical for protecting the lungs by managing airway inflammation.

## Figures and Tables

**Table 1:** Cytological analysis of bronchoalveolar lavage fluid.

	Experimental Groups					
	Vehicle	OVA	OVA + DEXA	OVA + AsE-100	OVA + AsE-200	OVA + AsE-400
Total cells	16.60 ± 2.72	73.00 ± 8.34 ^a^	28.60 ± 6.62 ^a,c^	49.90 ± 6.40 ^a,c,e^	38.70 ± 5.87 ^a,c,e^	30.10 ± 4.53 ^a,c^
Total leukocytes	11.80 ± 2.62	47.00 ± 7.87 ^a^	17.90 ± 3.03 ^a,c^	28.50 ± 4.86 ^a,c,e^	24.10 ± 5.09 ^a,c^	18.50 ± 2.55 ^a,c^
Lymphocytes	8.60 ± 2.88	32.10 ± 5.02 ^a^	11.70 ± 3.09 ^c^	19.20 ± 4.76 ^a,c,f^	16.40 ± 4.53 ^a,c^	12.10 ± 1.97 ^c^
Neutrophils	2.10 ± 0.99	9.30 ± 3.59 ^a^	3.90 ± 1.20 ^b,d^	5.30 ± 0.82 ^a^	4.40 ± 0.84 ^a,d^	4.10 ± 0.74 ^a,d^
Eosinophils	0.11 ± 0.17	1.86 ± 0.23 ^a^	0.69 ± 0.27 ^a,c^	1.16 ± 0.28 ^a,c,f^	1.01 ± 0.17 ^a,c^	0.72 ± 0.23 ^a,c^
Monocytes	0.17 ± 0.13	3.10 ± 0.73 ^a^	0.79 ± 0.42 ^b,c^	1.99 ± 0.09 ^a,d,e^	1.62 ± 0.42 ^a,c,e^	0.86 ± 0.44 ^a,c^

All numerical values indicate ×10^4^ cells/mL. ^a^ *p* < 0.01, ^b^ *p* < 0.05 versus vehicle group; ^c^ *p* < 0.01, ^d^ *p* < 0.05 versus OVA group; ^e^ *p* < 0.01, ^f^ *p* < 0.05 versus OVA + DEXA group.

## Data Availability

Data are contained within the article and Supplementary Materials.

## Supplementary Materials

The following supporting information can be downloaded at: https://www.mdpi.com/article/10.3390/antiox12040922/s1, Figure S1: Original images for immunoblots.
