# Supplementary material for: Adenophora Stricta Root Extract Alleviates Airway Inflammation in Mice with Ovalbumin-Induced Allergic Asthma
Source: Antioxidants (Basel). 2023 Apr 13;12(4):922. doi: 10.3390/antiox12040922 (PMC10135616; doi:10.3390/antiox12040922)
Supplement: Supplementary file 1 [file antioxidants-12-00922-s001.zip › antioxidants-2332555-supplementary.pdf]

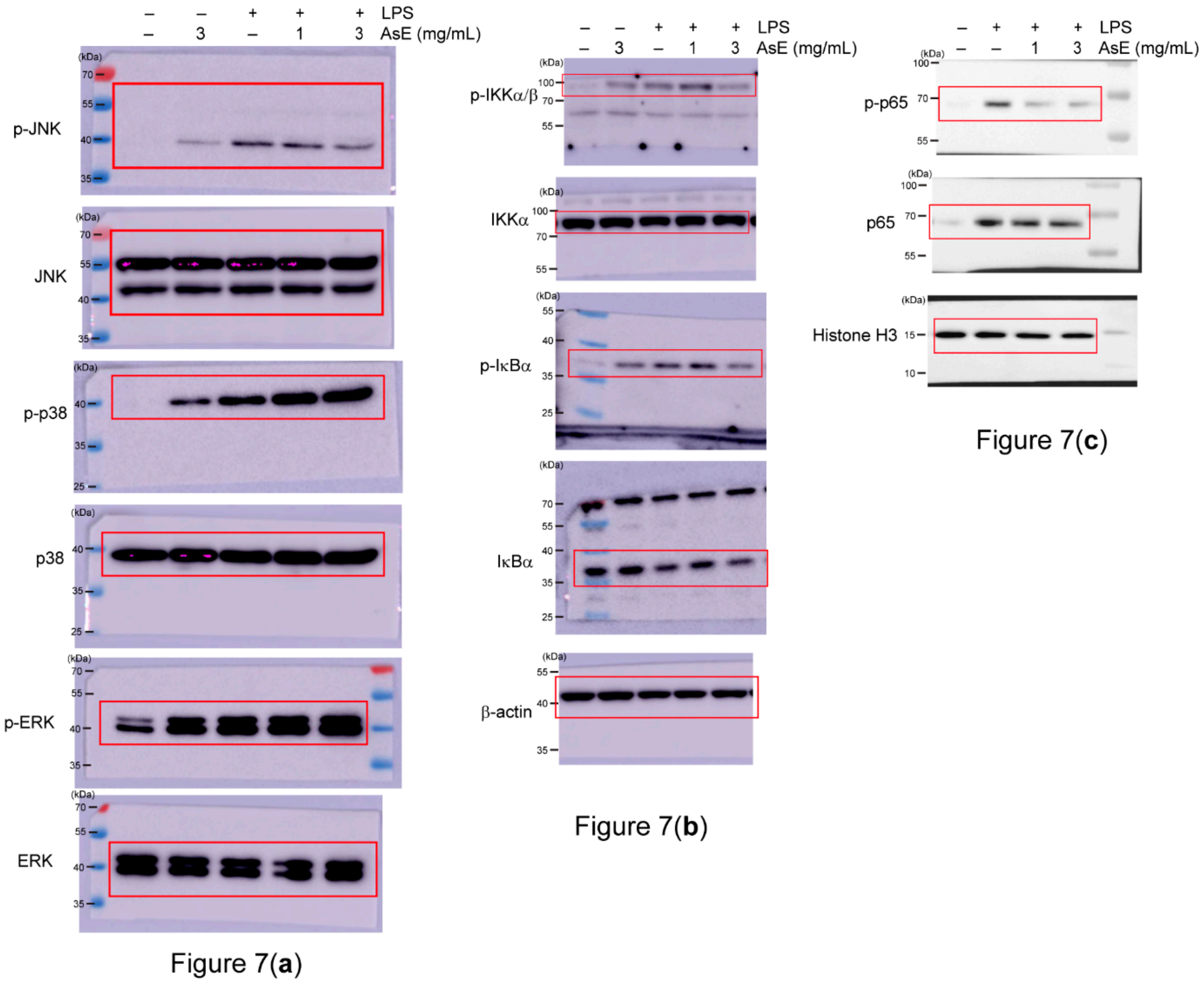


**Figure S1.** Original images for immunoblots. Red rectangles in original immunoblot images were cropped and used for figures.
